# Earth Poultices

**Published:** 1874-04

**Authors:** J. H. Payne

**Affiliations:** Preston, Mississippi


Earth Poultices.—Much is now being written about earth poultices, and the experience of all who have tried them attest their value as curative, and yet they are not new remedies, but old ones come into fashion again. It has been in domestic use for many years.

The following case occured in this county in 1844: Mr. Sulivan, a stout, healthy, plethoric man, was bitten on the foot by a very large rattle-snake, he was near a small brook and with his hoe dug a hole in the soft boggy earth, placed the limb up to the knee in it and drewing the wet earth tightly around it, he kept it in the earth twelve hours. He said he suffered very little pain, there was no sloughing or swelling, but said "it felt dead" for several days.

Perhaps the earth acted as a ligature around the leg, and the poison may have passed out by exosmosis.

My own experience with earth dressings may be worth recording, though the mode of preparing it, adopted by myself, differs from that usually pursued.

It has been used by myself and other members of the profession, to whom I have recommended it, with the most gratifying results, in the following cases:

Ulcers, old and indolent, recent wounds, painful and imflamed, gunshot wounds, fresh wounds, etc. Where the secretion of pus was superabundant and unhealthy, it was reduced in a short time to a normal standard in quantity and character, the pain subsiding with this change.

℞. Carbolic acid one part; boiled linseed oil four parts; red and yellow clay earth, in fine powder. Pour enough of the carb. oil on the powdered earth to make a soft consistent paste, spread this half an inch thick on cloth and apply to diseased parts, letting the paste extend over the inflamed skin around the ulcer or wound. Where the ulcer or wound is deep, I usually pour some of the oil on lint and fill up the cavity with it, as the earth is sometimes a little troublesome to remove from such places.

Common red earth, is composed principally of silex, aluminum and iron. How this combination acts upon the diseased capillaries and exposed nerve filaments I will not pretend to say, perhaps the carbolic acid acts an important part. Be this as it may, one thing I do know, the combination has proven a most precious remedy in my hands, and I will hold to it until a better can be found.

J. H. PAYNE.

PRESTON, MISSISSIPPI.

